# Efficacy of Prednisone Avoidance in Patients With Liver Transplant Using the U.S. Food and Drug Administration Adverse Event Reporting System

**DOI:** 10.7759/cureus.60193

**Published:** 2024-05-13

**Authors:** Toru Ogura, Chihiro Shiraishi

**Affiliations:** 1 Clinical Research Support Center, Mie University Hospital, Tsu, JPN; 2 Department of Pharmacy, Mie University Hospital, Tsu, JPN

**Keywords:** control for confounding, database, immunosuppressant combination, large sample size, reporting odds ratio, transplant rejection

## Abstract

Background

Immunosuppressants are administered in various combinations to prevent immune-induced transplant rejection in patients with liver transplant, as each immunosuppressant acts on different cellular sites. However, the use of multiple immunosuppressants also increases the risk for adverse events. Therefore, it is desirable to reduce the types of immunosuppressants administered without increasing the incidence of transplant rejection. The effectiveness of prednisone avoidance has been suggested, although this was not based on statistical significance in many instances. To definitively establish the effectiveness of prednisone avoidance, a statistically significant difference from a prednisone-use group should be demonstrated. Additionally, the effectiveness of prednisone avoidance might vary depending on the combination of other immunosuppressants administered. It has therefore been considered necessary to investigate, for various immunosuppressant combinations, the administration patterns in which prednisone avoidance is effective.

Objectives

This study aimed to investigate the effectiveness of prednisone avoidance in patients with liver transplant and discuss the results based on statistically significant differences.

Methods

Data from the U.S. Food and Drug Administration Adverse Event Reporting System (FAERS) were obtained. In studying immunosuppressant combinations, it was essential to control for confounding. Thus, the immunosuppressant combinations, excluding prednisone, were kept the same in the two groups being compared (prednisone-use and prednisone-avoidance groups). The large sample from FAERS allowed for those various immunosuppressant combinations to be compared. Comparisons of transplant rejection in the prednisone-use and prednisone-avoidance groups used the reporting odds ratio (ROR) and the adjusted ROR (aROR), which controlled for differences in patient background.

Results

With the prednisone-use groups being set as the reference, ROR and aROR were calculated for the prednisone-avoidance groups. Various immunosuppressant combinations were evaluated, and in four patterns - (1) the combination of prednisone and tacrolimus, (2) the combination of prednisone, cyclosporine, and tacrolimus, (3) the combination of prednisone, tacrolimus, and basiliximab, and (4) the combination of prednisone and everolimus) - both the ROR and the aROR for transplant rejection in the prednisone-avoidance group were significantly <1.000.

Conclusions

This study identified effective immunosuppressant combinations for prednisone avoidance that were not associated with increased transplant rejection. The evidence supporting the effectiveness of prednisone avoidance is strengthened when combined with results from previous studies.

## Introduction

Patients with liver transplant may experience immunity-driven transplant rejection. Immunosuppressants have been developed to prevent such occurrences, and nine main types, including prednisone (including prednisolone and methylprednisolone) [1], cyclosporine [1], tacrolimus [1], mycophenolate mofetil [1], everolimus [2], azathioprine [1], rituximab [3], mizoribine [2], and basiliximab [1], are currently on the market. Immunosuppressants are often administered in combination to enhance the effectiveness, but the use of multiple immunosuppressants also increases the risk for adverse events. Various researchers have therefore conducted studies on how to minimize, withdraw, and avoid the use of immunosuppressants [4]. Of those studies, some have aimed to validate prednisone avoidance by comparing the incidences of transplant rejection in prednisone-use and prednisone-avoidance groups [5-7]. Several studies observed a lower incidence of transplant rejection in the prednisone-avoidance group than in the prednisone-use group; however, none of the differences were often statistically significant. Nevertheless, the authors concluded that prednisone avoidance is effective. To definitively establish the effectiveness of prednisone avoidance, a statistically significant difference from a prednisone-use group should be demonstrated. Moreover, the effectiveness of prednisone avoidance might vary depending on the combination of other immunosuppressants administered. A limitation of previous clinical trials was the difficulty in obtaining a large sample, leading to comparisons between just two groups: a specific prednisone-use group and a specific prednisone-avoidance group. It has therefore been considered necessary to investigate, for various immunosuppressant combinations, the patterns of administration in which prednisone avoidance is effective. However, investigating many immunosuppressant combinations in a clinical trial has been impractical given the large sample required.

One previous clinical trial compared tacrolimus alone with tacrolimus, azathioprine, and prednisone used together [6]. The study design, which lacked control for confounding [8], did not allow for a determination of whether prednisone avoidance, cyclosporine avoidance, or both prednisone avoidance and cyclosporine avoidance were effective. If the purpose was to investigate prednisone avoidance, one of two study designs is appropriate: a comparison of tacrolimus alone with tacrolimus and prednisone used together, or a comparison of tacrolimus and azathioprine used together with tacrolimus, azathioprine, and prednisone used together.

To overcome those difficulties, data for the present study were obtained from the U.S. Food and Drug Administration Adverse Event Reporting System (FAERS) [9]. The FAERS database records adverse events reported worldwide. FAERS data include the name of the drug administered, the reason for its administration, and the name of the adverse event. FAERS data have been used to investigate the occurrence of adverse events after immunosuppressant administration not only to patients with liver transplant but also to patients with various other organ transplants [10,11].

Adverse events known to occur after prednisone administration include blood glucose increased, neuropsychiatric symptoms, hirsutism, leukocytosis, dyslipidemia, blood pressure increased, increased appetite, gastric ulcers, and cataract [1,12,13]. Some of the known adverse events for other immunosuppressants resemble those of prednisone, but each immunosuppressant also has unique adverse events. Cyclosporine or tacrolimus administration can trigger increased blood potassium, increased blood uric acid, liver disorder, and diabetes mellitus [1,13,14]. Mycophenolate mofetil can trigger diarrhea, anemia, anorexia, and pyrexia [1,13].

## Materials and methods

Data source

FAERS is an unlinkable, anonymized database that has been made publicly available on a quarterly basis since January 2004. The database began as the Adverse Event Reporting System (AERS) for the first quarter of 2004 (2004Q1) and continued until 2012Q3. The FAERS database, which contains more data elements than the AERS database, replaced AERS in 2012Q4. For this study, AERS (aers_ascii_yyyyQq.zip) and FAERS (faers_ascii_yyyyQq.zip) data files (where yyyy and q represent the year and quarter, respectively) were downloaded on March 3, 2024. Differences between the AERS and FAERS data elements were noted and addressed based on their descriptions. All subsequent references to FAERS data therefore include AERS data. FAERS data are provided in seven files, of which five were included in our analysis: patient demographic and administrative information (DEMOyyQq.txt, where yy represents the last two digits of the year), drug information (DRUGyyQq.txt), adverse event information (REACyyQq.txt), drug therapy start and end dates (THERyyQq.txt), and indications for use (INDIyyQq.txt). When new information is added to the existing data in FAERS, the existing data in the database are updated by incrementing the safety report version number {caseversion} rather than by overwriting. Throughout this study, names of the data elements used in FAERS are indicated using the curly braces convention. Only the highest {caseversion} number was used. In the AERS data, {caseversion} was not provided; however, this judgment could be made using the case identification number {CASE}. Data-handling, such as adjusting the unit of age to years, the unit of weight to kilograms, and responding to unexpected inputs, was required when using {sex}, patient's age at the adverse event {age}, {weight}, and country of the reporter {reporter_country} for statistical analyses.

Approval from an institutional review board was not required because the FAERS is an unlinkable, anonymized database that is open to the public.

Study design

Two inclusion criteria were set before the start of the study: (1) patients with liver transplant and (2) patients administered one or more of the nine immunosuppressants of interest between 2004Q1 and 2023Q4. In the FAERS data between 2014Q3 and 2023Q4, immunosuppressants were identified by trade name using the variable for the product’s active ingredient, {prod_ai}. The {prod_ai} was not provided between 2004Q1 and 2014Q2, and therefore for those periods, immunosuppressants had to be identified by both trade and brand names using the variable for the medical product, {drugname}. Of the database records extracted for the specified immunosuppressants, those records in which the data element describing the indication for use, {indi_pt}, was liver transplantation were retained.

Three exclusion criteria were also set before the start of the study: (1) an immunosuppressant had never been administered before the adverse event occurred, (2) prednisone use or prednisone avoidance had been determined by the presence of another condition, and (3) patients with multiple organ transplants. Thus, if a patient had received immunosuppressants both before and after the adverse event occurred, data for immunosuppressants administered only after the adverse event occurred were excluded under the first criterion, whereas data for immunosuppressants administered before the adverse event occurred were retained. Exclusion or retention was determined based on the date that the specific immunosuppressant was started (or re-started), {start_dt}, the date on which the adverse event occurred or began, {event_dt}, and the date on which the specific immunosuppressant was stopped, {end_dt}. Because the sample became smaller when case records with missing data for those three dates were excluded, only case records for which the immunosuppressant could be reliably judged to have been started after the adverse event occurred were excluded. Patients who were eligible for inclusion in the prednisone-use group, but who had conditions such as ABO-incompatible transplant [15], sclerosing cholangitis [16], and autoimmune hepatitis [17] and thus did not have the option of prednisone avoidance, were excluded under the second criterion. Patients who were eligible for inclusion in the prednisone-avoidance group, but who had conditions such as hepatitis B [18] and hepatitis C [18] and thus did not have the option of prednisone use, were similarly excluded. These conditions were identified based on all drugs administered to the particular patient, {indi_pt}. If a patient had undergone organ transplantation other than liver transplantation, {indi_pt}, that patient record was excluded under the third criterion, as prednisone avoidance is often not a viable option in such patients.

The endpoint was the occurrence or nonoccurrence of transplant rejection. The Medical Dictionary for Regulatory Activities, {pt}, provided the preferred terms to describe adverse events. Given that our study focused on patients with liver transplant, adverse events reported as either transplant rejection or liver transplant rejection were considered identical. FAERS collects reports of adverse events that occur after drug administration, which are often considered drug-related adverse events. However, when adverse events occur after administration of a prophylactic drug, they can be considered to be adverse events that could not have been prevented by that prophylactic drug. Previous studies have used this data element in FAERS to evaluate the effectiveness of preventive drugs [11]. Transplant rejection was thus considered not to be a drug-related adverse event but rather a transplant-related adverse event that occurred because administration of the immunosuppressant could not prevent the event.

Groups were established based on immunosuppressant combinations. These groups were identified using nine-digit binary numbers. The use (binary 1) or nonuse (binary 0) of each immunosuppressant was assigned for (left to right) (1) prednisone, (2) cyclosporine, (3) tacrolimus, (4) mycophenolate mofetil, (5) everolimus, (6) azathioprine, (7) rituximab, (8) mizoribine, and (9) basiliximab. Thus, the 100000000 group consisted of patients who were administered prednisone and none of the other eight immunosuppressants. When considering immunosuppressant combinations, controlling for confounders is essential. Methods such as randomization, restriction, and matching are known to mitigate confounding [8]. Because of the large sample in this study, restriction was used to control for confounding. Confounding was controlled by specifying that XXXXXXXX, the immunosuppressants other than prednisone, were to be the same for the 1XXXXXXXX (prednisone-use) and 0XXXXXXXX (prednisone-avoidance) groups.

Statistical analysis

Continuous and categorical data are summarized as median with first and third quartiles and as frequency and reporting proportion (RP) [19], respectively. The RP was calculated as (number of patients in the category of interest) / (number of patients in the target group) × 100. The reporting odds ratio (ROR) [20,21] and its 95% confidence interval (CI) and the adjusted ROR (aROR) and its 95% CI were calculated using univariate and multivariate binomial logistic regression analyses, respectively. Because the United States had the highest number of reports, the univariate and multivariate binomial logistic regression analyses used {reporter_country} as binary data: the United States and other countries. The references for the ROR and the aROR were set to the 1XXXXXXXX group. Any adjustment elements used in the aROR were determined through variable selection in multivariate binomial logistic regression analysis. A p-value of <0.05 was considered statistically significant. The software R version 4.2.2 (R Foundation for Statistical Computing, Vienna, Austria) was used for statistical analyses.

Calculating the incidence of each adverse event was impossible because the FAERS database contains no records with zero adverse events. Therefore, as in earlier studies, this study also used the RP and ROR with the addition of “reporting” to differentiate the statistical analysis methods using FAERS data from the usual statistical analysis methods.

## Results

Patient background

**Figure 1:**
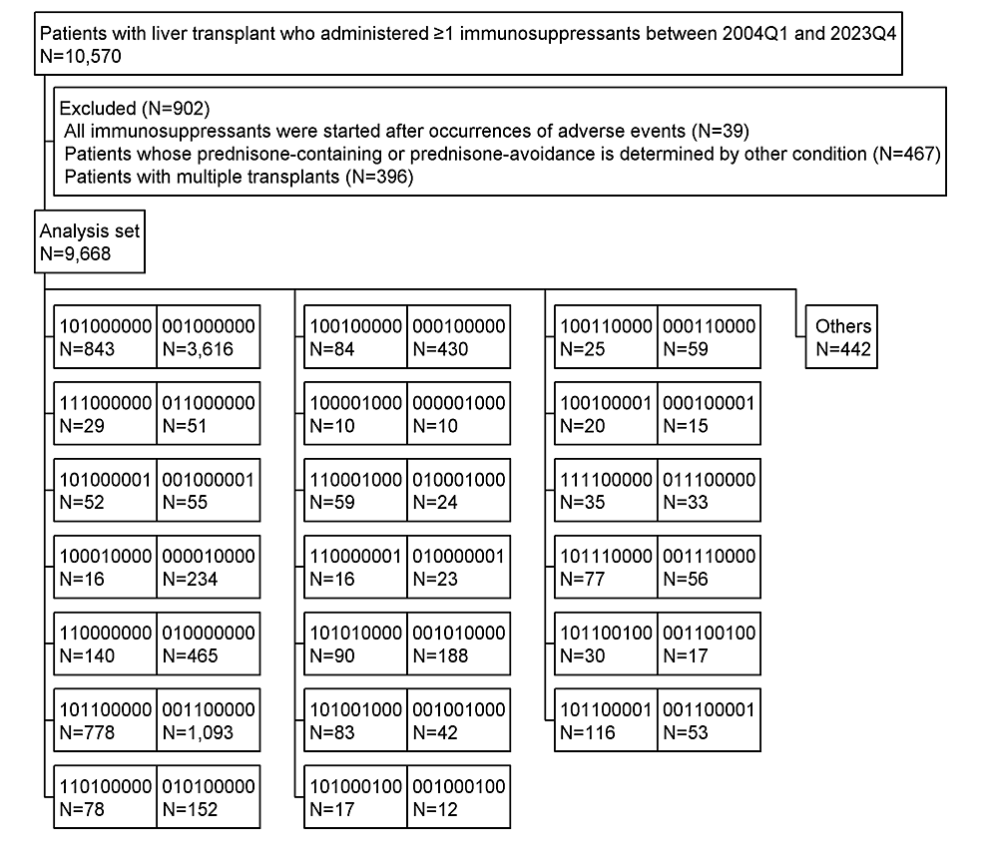
Flowchart of patients with liver transplant who were administered one or more immunosuppressants. Groups were established based on immunosuppressant combinations, with each group represented by a nine-digit binary number. In these nine-digit binary numbers, the use and nonuse of each immunosuppressant were indicated by 1 and 0, respectively. The digits from the first to the ninth (from left to right) corresponded to (1) prednisone, (2) cyclosporine, (3) tacrolimus, (4) mycophenolate mofetil, (5) everolimus, (6) azathioprine, (7) rituximab, (8) mizoribine, and (9) basiliximab, respectively. When at least one of side-by-side groups had N<10, they were grouped together as "others."

A total of 10,570 patients who had undergone liver transplant and who had been administered one or more immunosuppressants between 2004Q1 and 2023Q4 were identified. After 902 had been excluded under the predetermined exclusion criteria, 9,668 patients were included in the analysis set. Figure 1 presents a side-by-side sample of the 1XXXXXXXX (prednisone-use) group and the 0XXXXXXXX (prednisone-avoidance) group. This study targeted groups in which the side-by-side samples both consisted of N≥10. When at least one of the side-by-side groups consisted of N<10, both sets of patients were grouped together as “others.” Table 1 summarizes the patient background of the 12 groups discussed in the main text.

**Table 1:** Summary of patients' background. Age and weight are summarized as median with first and third quartiles. Other data are summarized as frequency (RP). Groups were established based on immunosuppressant combinations, with each group represented by a nine-digit binary number. In these nine-digit binary numbers, the use and nonuse of each immunosuppressant were indicated by 1 and 0, respectively. The digits from the first to the ninth (from left to right) corresponded to (1) prednisone, (2) cyclosporine, (3) tacrolimus, (4) mycophenolate mofetil, (5) everolimus, (6) azathioprine, (7) rituximab, (8) mizoribine, and (9) basiliximab, respectively. Q1, first quartile; Q3, third quartile; RP, reporting proportion

	101000000	001000000	111000000	011000000	101000001	001000001	100010000	000010000	110000000	010000000	101100000	001100000
	N=843	N=3,616	N=29	N=51	N=52	N=55	N=16	N=234	N=140	N=465	N=778	N=1,093
Sex												
Female, n (RP)	279 (33.1)	1,344 (37.2)	12 (41.4)	26 (51.0)	24 (46.2)	28 (50.9)	4 (25.0)	66 (28.2)	55 (39.3)	181 (38.9)	266 (34.2)	361 (33.0)
Male, n (RP)	453 (53.7)	1,965 (54.3)	14 (48.3)	20 (39.2)	16 (30.8)	21 (38.2)	10 (62.5)	133 (56.8)	56 (40.0)	248 (53.3)	409 (52.6)	629 (57.5)
Unknown, n (RP)	111 (13.2)	307 (8.5)	3 (10.3)	5 (9.8)	12 (23.1)	6 (10.9)	2 (12.5)	35 (15.0)	29 (20.7)	36 (7.7)	103 (13.2)	103 (9.4)
Age												
Median	51.0	55.0	44.0	40.5	45.5	8.5	57.0	60.0	51.0	55.0	55.0	54.0
Q1-Q3	23.0-59.0	37.0-63.0	25.0-62.5	20.2-60.2	11.0-57.2	1.8-48.5	40.0-66.0	54.0-66.0	30.0-61.0	42.0-64.0	42.0-62.0	41.0-62.0
Unknown, n (RP)	161 (19.1)	1,080 (29.9)	2 (6.9)	11 (21.6)	12 (23.1)	11 (20.0)	5 (31.2)	90 (38.5)	35 (25.0)	130 (28.0)	155 (19.9)	239 (21.9)
Weight, kg												
Median	60.0	68.1	60.5	70.8	40.5	15.5	70.0	68.5	70.8	71.0	73.0	73.0
Q1-Q3	45.0-72.6	55.0-88.0	45.2-64.7	47.2-72.9	40.2-40.8	12.1-72.8	65.0-74.5	59.5-84.8	45.6-92.5	59.0-80.0	57.7-83.0	58.8-96.0
Unknown, n (RP)	667 (79.1)	3,121 (86.3)	25 (86.2)	45 (88.2)	50 (96.2)	43 (78.2)	9 (56.2)	192 (82.1)	112 (80.0)	364 (78.3)	665 (85.5)	918 (84.0)
Country												
United States, n (RP)	247 (29.3)	1,956 (54.1)	4 (13.8)	16 (31.4)	2 (3.8)	3 (5.5)	2 (12.5)	66 (28.2)	23 (16.4)	137 (29.5)	233 (29.9)	345 (31.6)
France, n (RP)	64 (7.6)	165 (4.6)	1 (3.4)	0 (0.0)	0 (0.0)	10 (18.2)	0 (0.0)	23 (9.8)	12 (8.6)	24 (5.2)	94 (12.1)	199 (18.2)
China, n (RP)	32 (3.8)	284 (7.9)	3 (10.3)	1 (2.0)	7 (13.5)	1 (1.8)	0 (0.0)	0 (0.0)	0 (0.0)	11 (2.4)	71 (9.1)	66 (6.0)
Germany, n (RP)	34 (4.0)	78 (2.2)	2 (6.9)	16 (31.4)	4 (7.7)	7 (12.7)	4 (25.0)	27 (11.5)	34 (24.3)	32 (6.9)	22 (2.8)	43 (3.9)
Japan, n (RP)	89 (10.6)	147 (4.1)	4 (13.8)	4 (7.8)	3 (5.8)	1 (1.8)	0 (0.0)	10 (4.3)	7 (5.0)	22 (4.7)	35 (4.5)	23 (2.1)
Spain, n (RP)	22 (2.6)	100 (2.8)	1 (3.4)	1 (2.0)	1 (1.9)	8 (14.5)	1 (6.2)	15 (6.4)	14 (10.0)	20 (4.3)	30 (3.9)	72 (6.6)
Italy, n (RP)	37 (4.4)	113 (3.1)	2 (6.9)	0 (0.0)	14 (26.9)	5 (9.1)	3 (18.8)	6 (2.6)	8 (5.7)	51 (11.0)	8 (1.0)	31 (2.8)
United Kingdom, n (RP)	40 (4.7)	85 (2.4)	0 (0.0)	0 (0.0)	3 (5.8)	0 (0.0)	0 (0.0)	1 (0.4)	1 (0.7)	15 (3.2)	59 (7.6)	33 (3.0)
Canada, n (RP)	30 (3.6)	127 (3.5)	2 (6.9)	1 (2.0)	0 (0.0)	0 (0.0)	0 (0.0)	0 (0.0)	8 (5.7)	11 (2.4)	19 (2.4)	28 (2.6)
Colombia, n (RP)	34 (4.0)	77 (2.1)	0 (0.0)	2 (3.9)	0 (0.0)	0 (0.0)	1 (6.2)	1 (0.4)	0 (0.0)	0 (0.0)	19 (2.4)	9 (0.8)
Others, n (RP)	161 (19.1)	352 (9.7)	9 (31.0)	6 (11.8)	17 (32.7)	14 (25.5)	4 (25.0)	53 (22.6)	13 (9.3)	73 (15.7)	154 (19.8)	190 (17.4)
Unknown, n (RP)	53 (6.3)	132 (3.7)	1 (3.4)	4 (7.8)	1 (1.9)	6 (10.9)	1 (6.2)	32 (13.7)	20 (14.3)	69 (14.8)	34 (4.4)	54 (4.9)

Adverse events

**Figure 2:**
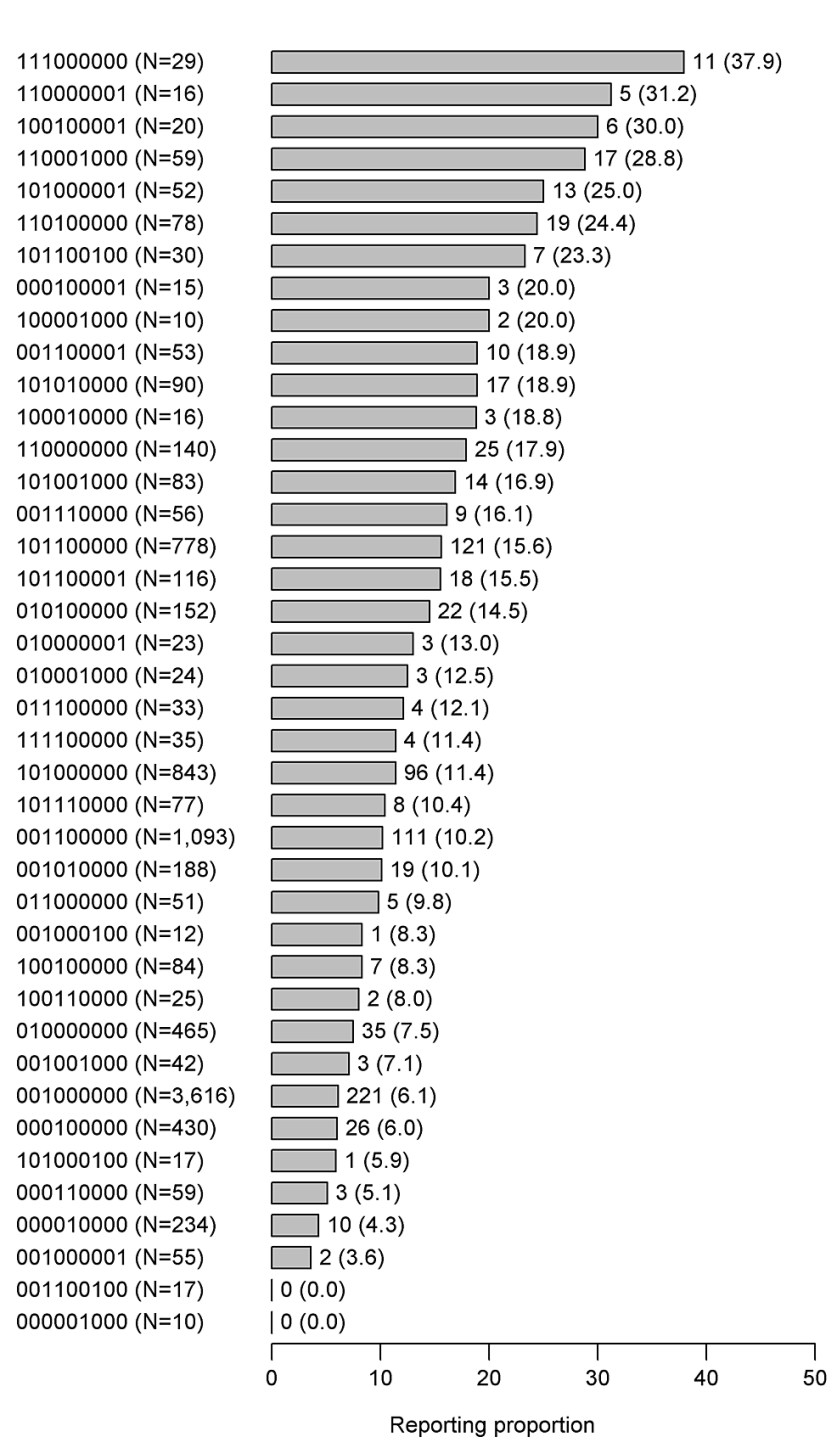
Descending order of the RP of transplant rejection by group. Data are summarized as frequency (reporting proportion). Groups were established based on immunosuppressant combinations, with each group represented by a nine-digit binary number. In these nine-digit binary numbers, the use and nonuse of each immunosuppressant were indicated by 1 and 0, respectively. The digits from the first to the ninth (from left to right) corresponded to (1) prednisone, (2) cyclosporine, (3) tacrolimus, (4) mycophenolate mofetil, (5) everolimus, (6) azathioprine, (7) rituximab, (8) mizoribine, and (9) basiliximab, respectively. RP, reporting proportion

Figure 2 presents a bar graph of the RP for transplant rejection, by group, in descending order. The RP of the 111000000 group was 37.9, which was the highest among all groups. The top seven positions were all occupied by 1XXXXXXXX (prednisone-use) groups. Table 2 presents the ROR and the aROR. When the 101000000 group was set as the reference, both the ROR and the aROR of the 001000000 group were significantly <1.000 (ROR: 0.507 [95% CI: 0.394-0.652], p < 0.001; aROR: 0.600 [95% CI: 0.449-0.803], p = 0.001). When the 111000000 group was set as the reference, the ROR of the 011000000 group was significantly <1.000 (ROR: 0.178 [95% CI: 0.054-0.584], p = 0.004), and the aROR was the same as the ROR because all adjustment variables were not selected with p≥0.05. When the 101000001 group was set as the reference, both the ROR and aROR of the 001000001 group were significantly <1.000 (ROR: 0.113 [95% CI: 0.024-0.531], p = 0.006; aROR: 0.116 [95% CI: 0.023-0.591], p = 0.010). When the 100010000 group was set as the reference, the ROR of the 000010000 group was significantly <1.000 (ROR: 0.193 [95% CI: 0.047-0.789], p = 0.022), and the aROR was the same as the ROR because all adjustment variables were not selected with p≥0.05. When the 110000000 group was set as the reference, the ROR of the 010000000 group was significantly <1.000 (ROR: 0.374 [95% CI: 0.215-0.651], p < 0.001) but the aROR of the 010000000 group was not statistically significant (aROR: 0.824 [95% CI: 0.408-1.664], p = 0.559). When the 101100000 group was set as the reference, the ROR of the 001100000 group was significantly <1.000 (ROR: 0.614 [95% CI: 0.466-0.809], p = 0.001), but the aROR of the 001100000 group was not statistically significant (aROR: 0.787 [95% CI: 0.570-1.088], p = 0.148). Table 3 shows the RP of each adverse event by group.

**Table 2:** The ROR and aROR of transplant rejection. Groups were established based on immunosuppressant combinations, with each group represented by a nine-digit binary number. In these nine-digit binary numbers, the use and nonuse of each immunosuppressant were indicated by 1 and 0, respectively. The digits from the first to the ninth (from left to right) corresponded to (1) prednisone, (2) cyclosporine, (3) tacrolimus, (4) mycophenolate mofetil, (5) everolimus, (6) azathioprine, (7) rituximab, (8) mizoribine, and (9) basiliximab, respectively. –: Not calculable (because the frequency of adverse event occurrence was 0). aROR, adjusted reporting odds ratio; CI, confidence interval; ROR, reporting odds ratio

Combination	Variable	ROR (95%CI)	p-value	aROR (95%CI)	p-value
101000000	Prednisone avoidance	0.507 (0.394-0.652)	<0.001	0.600 (0.449-0.803)	0.001
	Male	0.766 (0.597-0.982)	0.035		
	Age	0.992 (0.986-0.997)	0.003	0.993 (0.987-0.998)	0.011
	Weight	0.986 (0.978-0.995)	0.002		
	United States	1.326 (0.834-2.110)	0.233		
111000000	Prednisone avoidance	0.178 (0.054-0.584)	0.004	0.178 (0.054-0.584)	0.004
	Male	0.804 (0.248-2.608)	0.716		
	Age	0.986 (0.962-1.011)	0.280		
	Weight	1.009 (0.944-1.078)	0.787		
	United States	0.541 (0.061-4.767)	0.580		
101000001	Prednisone avoidance	0.113 (0.024-0.531)	0.006	0.116 (0.023-0.591)	0.010
	Male	0.104 (0.013-0.842)	0.034	0.093 (0.011-0.786)	0.029
	Age	0.996 (0.971-1.022)	0.763		
	Weight	-	-		
	United States	6.385 (0.826-49.362)	0.076		
100010000	Prednisone avoidance	0.193 (0.047-0.789)	0.022	0.193 (0.047-0.789)	0.022
	Male	0.307 (0.084-1.126)	0.075		
	Age	0.993 (0.944-1.045)	0.793		
	Weight	0.975 (0.929-1.024)	0.320		
	United States	-	-		
110000000	Prednisone avoidance	0.374 (0.215-0.651)	<0.001	0.824 (0.408-1.664)	0.589
	Male	0.796 (0.432-1.467)	0.465		
	Age	0.983 (0.969-0.997)	0.014	0.983 (0.970-0.997)	0.020
	Weight	0.995 (0.971-1.020)	0.708		
	United States	0.312 (0.042-2.341)	0.257		
101100000	Prednisone avoidance	0.614 (0.466-0.809)	0.001	0.787 (0.570-1.088)	0.148
	Male	0.833 (0.610-1.139)	0.252		
	Age	0.990 (0.982-0.999)	0.022	0.990 (0.982-0.999)	0.021
	Weight	0.985 (0.969-1.000)	0.052		
	United States	1.304 (0.631-2.692)	0.473		

**Table 3:** Summary of each adverse event by group. Data are summarized as frequency (reporting proportion). If multiple adverse events are reported in a patient, each adverse event is counted. Groups were established based on immunosuppressant combinations, with each group represented by a nine-digit binary number. In these nine-digit binary numbers, the use and nonuse of each immunosuppressant were indicated by 1 and 0, respectively. The digits from the first to the ninth (from left to right) corresponded to (1) prednisone, (2) cyclosporine, (3) tacrolimus, (4) mycophenolate mofetil, (5) everolimus, (6) azathioprine, (7) rituximab, (8) mizoribine, and (9) basiliximab, respectively.

Adverse event name	101000000	001000000	111000000	011000000	101000001	001000001	100010000	000010000	110000000	010000000	101100000	001100000
	N=843	N=3,616	N=29	N=51	N=52	N=55	N=16	N=234	N=140	N=465	N=778	N=1,093
Transplant rejection	97 (11.5)	224 (6.2)	11 (37.9)	5 (9.8)	13 (25.0)	2 (3.6)	3 (18.8)	10 (4.3)	25 (17.9)	35 (7.5)	123 (15.8)	112 (10.2)
Blood glucose increased	1 (0.1)	18 (0.5)	0 (0.0)	1 (2.0)	0 (0.0)	0 (0.0)	0 (0.0)	4 (1.7)	0 (0.0)	1 (0.2)	12 (1.5)	4 (0.4)
Blood pressure increased	1 (0.1)	14 (0.4)	0 (0.0)	1 (2.0)	0 (0.0)	0 (0.0)	0 (0.0)	0 (0.0)	1 (0.7)	0 (0.0)	1 (0.1)	6 (0.5)
Cataract	1 (0.1)	3 (0.1)	0 (0.0)	0 (0.0)	0 (0.0)	0 (0.0)	0 (0.0)	0 (0.0)	0 (0.0)	1 (0.2)	1 (0.1)	0 (0.0)
Dyslipidemia	0 (0.0)	2 (0.1)	1 (3.4)	0 (0.0)	0 (0.0)	0 (0.0)	0 (0.0)	0 (0.0)	0 (0.0)	0 (0.0)	2 (0.3)	4 (0.4)
Gastric ulcer	2 (0.2)	0 (0.0)	0 (0.0)	0 (0.0)	0 (0.0)	0 (0.0)	0 (0.0)	0 (0.0)	0 (0.0)	1 (0.2)	1 (0.1)	0 (0.0)
Hirsutism	0 (0.0)	0 (0.0)	0 (0.0)	0 (0.0)	0 (0.0)	0 (0.0)	0 (0.0)	0 (0.0)	1 (0.7)	1 (0.2)	0 (0.0)	0 (0.0)
Increased appetite	0 (0.0)	0 (0.0)	0 (0.0)	0 (0.0)	0 (0.0)	0 (0.0)	0 (0.0)	0 (0.0)	0 (0.0)	0 (0.0)	0 (0.0)	0 (0.0)
Leukocytosis	2 (0.2)	5 (0.1)	0 (0.0)	0 (0.0)	0 (0.0)	0 (0.0)	0 (0.0)	0 (0.0)	0 (0.0)	1 (0.2)	2 (0.3)	2 (0.2)
Neurological symptom	4 (0.5)	4 (0.1)	0 (0.0)	0 (0.0)	0 (0.0)	0 (0.0)	0 (0.0)	0 (0.0)	0 (0.0)	0 (0.0)	1 (0.1)	1 (0.1)
Psychiatric symptom	1 (0.1)	3 (0.1)	0 (0.0)	1 (2.0)	0 (0.0)	0 (0.0)	0 (0.0)	0 (0.0)	1 (0.7)	0 (0.0)	0 (0.0)	0 (0.0)
Anemia	26 (3.1)	36 (1.0)	0 (0.0)	0 (0.0)	2 (3.8)	0 (0.0)	0 (0.0)	3 (1.3)	6 (4.3)	5 (1.1)	24 (3.1)	27 (2.5)
Anorexia	1 (0.1)	2 (0.1)	0 (0.0)	0 (0.0)	0 (0.0)	0 (0.0)	0 (0.0)	0 (0.0)	0 (0.0)	3 (0.6)	0 (0.0)	3 (0.3)
Arrhythmia	2 (0.2)	6 (0.2)	0 (0.0)	0 (0.0)	0 (0.0)	0 (0.0)	0 (0.0)	0 (0.0)	0 (0.0)	1 (0.2)	0 (0.0)	1 (0.1)
Blood potassium increased	0 (0.0)	9 (0.2)	0 (0.0)	0 (0.0)	0 (0.0)	0 (0.0)	0 (0.0)	0 (0.0)	0 (0.0)	2 (0.4)	1 (0.1)	3 (0.3)
Blood uric acid increased	0 (0.0)	3 (0.1)	0 (0.0)	0 (0.0)	0 (0.0)	0 (0.0)	0 (0.0)	0 (0.0)	0 (0.0)	2 (0.4)	1 (0.1)	0 (0.0)
Cardiomyopathy	2 (0.2)	6 (0.2)	0 (0.0)	0 (0.0)	0 (0.0)	0 (0.0)	0 (0.0)	0 (0.0)	1 (0.7)	0 (0.0)	0 (0.0)	0 (0.0)
Diabetes mellitus	11 (1.3)	46 (1.3)	0 (0.0)	2 (3.9)	0 (0.0)	0 (0.0)	0 (0.0)	2 (0.9)	3 (2.1)	7 (1.5)	5 (0.6)	23 (2.1)
Diarrhea	32 (3.8)	88 (2.4)	1 (3.4)	3 (5.9)	1 (1.9)	2 (3.6)	0 (0.0)	6 (2.6)	2 (1.4)	17 (3.7)	41 (5.3)	53 (4.8)
Gingival hyperplasia	0 (0.0)	1 (0.0)	0 (0.0)	0 (0.0)	0 (0.0)	0 (0.0)	0 (0.0)	0 (0.0)	2 (1.4)	0 (0.0)	0 (0.0)	0 (0.0)
Ileus	2 (0.2)	7 (0.2)	0 (0.0)	0 (0.0)	0 (0.0)	0 (0.0)	0 (0.0)	0 (0.0)	1 (0.7)	1 (0.2)	1 (0.1)	1 (0.1)
Leukoencephalopathy	2 (0.2)	6 (0.2)	0 (0.0)	0 (0.0)	1 (1.9)	0 (0.0)	0 (0.0)	0 (0.0)	0 (0.0)	4 (0.9)	0 (0.0)	3 (0.3)
Leukopenia	5 (0.6)	35 (1.0)	0 (0.0)	1 (2.0)	0 (0.0)	0 (0.0)	0 (0.0)	3 (1.3)	1 (0.7)	3 (0.6)	8 (1.0)	12 (1.1)
Liver disorder	8 (0.9)	19 (0.5)	0 (0.0)	0 (0.0)	0 (0.0)	0 (0.0)	0 (0.0)	2 (0.9)	0 (0.0)	3 (0.6)	5 (0.6)	5 (0.5)
Lymphadenopathy	11 (1.3)	6 (0.2)	1 (3.4)	0 (0.0)	1 (1.9)	0 (0.0)	0 (0.0)	0 (0.0)	0 (0.0)	2 (0.4)	8 (1.0)	6 (0.5)
Nausea	16 (1.9)	75 (2.1)	1 (3.4)	4 (7.8)	1 (1.9)	0 (0.0)	0 (0.0)	10 (4.3)	1 (0.7)	7 (1.5)	5 (0.6)	20 (1.8)
Pneumonia cytomegaloviral	4 (0.5)	4 (0.1)	0 (0.0)	0 (0.0)	0 (0.0)	1 (1.8)	0 (0.0)	0 (0.0)	0 (0.0)	0 (0.0)	1 (0.1)	0 (0.0)
Pyrexia	56 (6.6)	93 (2.6)	1 (3.4)	3 (5.9)	6 (11.5)	12 (21.8)	2 (12.5)	9 (3.8)	19 (13.6)	18 (3.9)	48 (6.2)	25 (2.3)

## Discussion

Immunosuppressants are administered in various combinations, as each type acts on different cellular sites [22]. However, immunosuppressant combinations also increase the risk for adverse events, and thus many studies are being conducted to minimize their use to the extent possible [4]. Approaches to minimizing immunosuppressant use include immunosuppressant avoidance [5-7], immunosuppressant withdrawal [23,24], and immunosuppressant reduction [23]. Immunosuppressant switching is also being investigated as an approach to lower the risk of adverse events [25,26]. The randomized clinical trials investigating whether those approaches are effective use transplant rejection as an endpoint. Although reports from some trials revealed a significant reduction in transplant rejection [26], the incidence of transplant rejection in many others was lower in the avoidance group but not statistically significant [5-7]. The reason for the lack of statistical significance was thought to be the difficulty in obtaining a sufficiently large sample. A detailed review of each trial suggested that there was a potential to obtain statistically significant results with a large sample. Because conducting the clinical trial with a large sample is impractical, this study was designed to use the FAERS database. Four patterns of effective immunosuppressant administration were observed prednisone avoidance without increasing the occurrence of transplant rejection. Three of those patterns included tacrolimus, supporting the results of previous studies that found lower transplant rejection in a prednisone-avoidance group of the prednisone and tacrolimus pattern [5-7]. Additionally, the RP for transplant rejection was often higher in the prednisone-use group than in the prednisone-avoidance group (Figure 2).

The safety of prednisone avoidance was evaluated based on the types of adverse events that occurred. Diabetes mellitus is a common long-term complication after liver transplantation [14]. Groups that were administered calcineurin inhibitors (tacrolimus or cyclosporine) tended to have a higher RP for diabetes mellitus, which accords with results from a previous study [14]. Diarrhea is a common adverse event after the administration of cyclosporine and tacrolimus [27]. Anemia is a known adverse event associated with tacrolimus and mycophenolate mofetil [28,29]. Previous studies have reported severe cold agglutinin hemolytic anemia and severe persistent anemia with immunosuppressant administration, requiring the need for careful monitoring of treated patients.

The main strength of our study was its large sample size, which allowed for the evaluation of statistically significant differences between groups receiving various immunosuppressant combinations; however, our study also has some limitations. First, the incidence of transplant rejection could not be calculated because FAERS data only report the occurrence of adverse events. Second, FAERS data are subject to notoriety bias. Third, FAERS data contained numerous missing data. Whether immunosuppressants had been switched could not be determined when date elements were missing. Fourth, dose data were not used because those data were sometimes missing, although the dose of immunosuppressants should be considered. Finally, the medical histories of the patients were unknown except for liver transplant. However, by using {indi_pt}, it might be possible to identify patients with multiple organ transplants and those at high risk for transplant rejection, such as those with an ABO-incompatible transplant [15], sclerosing cholangitis [16], autoimmune hepatitis [17], hepatitis B [18], and hepatitis C [18]. This study excluded such patients because prednisone use and prednisone avoidance were determined by other conditions. However, if information indicating high risk was missing, exclusion would not have been possible.

Despite the foregoing limitations, the FAERS database has the advantage of containing many reports spanning the globe. However, the level of evidence might still be low based solely on the results of adverse event reporting. The results of this study must therefore be interpreted in conjunction with results from previous studies. The effectiveness of prednisone avoidance demonstrated in this study could be used to strengthen the level of evidence in previous studies. Moreover, the results of this study could be used to narrow the selection of immunosuppressant combinations for investigation in future studies.

## Conclusions

Previous clinical trials accepted the effectiveness of prednisone avoidance based on a lower incidence of transplant rejection in prednisone-avoidance groups; however, the difference between prednisone use and prednisone avoidance was often not statistically significant. In contrast, this study demonstrated the following four effective patterns of immunosuppressant administration for prednisone avoidance that are based on statistically significant differences: prednisone avoidance from (1) the combination of prednisone and tacrolimus, (2) the combination of prednisone, cyclosporine, and tacrolimus, (3) the combination of prednisone, tacrolimus, and basiliximab, and (4) the combination of prednisone and everolimus. The evidence supporting the effectiveness of prednisone avoidance is strengthened when the results of this study are combined with results from previous studies.
